# Adolescent-onset depression is associated with altered social functioning into middle adulthood

**DOI:** 10.1038/s41598-022-22131-1

**Published:** 2022-10-15

**Authors:** Katherine Chang, Kate Ryan Kuhlman

**Affiliations:** 1 Department of Psychological Science, School of Social Ecology, University of California, Irvine, 4546 Social & Behavioral Sciences Gateway, Irvine, CA 92697 USA; 2 Cousins Center for Psychoneuroimmunology, Semel Institute for Neuroscience and Human Development, University of California, Los Angeles, Los Angeles, CA USA

**Keywords:** Human behaviour, Psychiatric disorders

## Abstract

Depression during sensitive periods of social development may have consequences that extend well beyond mental health, and far into adulthood. This study compared the social functioning of adults with adolescent-onset depression (ages 10–20) to those with adult-onset depression (ages 21+). Participants were 3,360 adults (67.2% female; ages 42 ± 15) who had experienced major depression. Adult functional outcomes were marital status, divorce, number of children, years of education, employment status, household income, dependency on welfare, and obesity. Participants with depression during adolescence were less likely to get married, have children, and more likely to have lower household incomes. Depression during adolescence may be associated with broader functional outcomes that impact individuals and society, and may be mitigated through intervention and effective policy.

## Introduction

### Adolescent-onset depression is associated with altered social functioning into middle adulthood

Major depressive disorder (MDD) is one of the leading causes of disability worldwide^1,2^, with a reported lifetime prevalence of one in six men and one in four women^3^. Symptom presentation varies by severity and individual, but the disorder involves the co-occurrence of feelings of sadness, loss of interest or pleasure, changes in weight and sleep, increased fatigue, feelings of worthlessness or guilt, and suicidal ideation^4^. Depression can be devastating to multiple facets of an individual’s life, extending beyond sheer emotional distress. Compared to healthy controls, individuals who are currently depressed have diminished academic achievement and occupational functioning, experience more interpersonal conflict, and are more likely to be afflicted with comorbid conditions^5–7^. The concurrence of multiple stressful life events with depression is termed stress generation, which expounds how the cycle of stress and depression affects an individual’s life and social world in a disruptive, reverberating fashion^8^. The present study explored whether onset of MDD during adolescence, a sensitive period of social development, is associated with poorer adulthood functioning in ways that extend beyond health.

During sensitive periods of psychosocial development^9^, the harmful effects of depression and other psychiatric disorders may be amplified. Adolescence is a sensitive and transitional period of development during which individuals undergo dramatic biological, cognitive, and social changes^10–12^. It typically starts with pubertal maturation (beginning around age 10) and ends when individuals take on independent social roles and adult responsibilities^12^. During this time, adolescents are learning to navigate an increasingly complex social world while more sensitive to social stimuli, social deprivation, and peer rejection^10,12,13^. Superimposing a syndrome that involves social withdrawal, amotivation, and profound changes to weight and sleep that have both visible and cognitive consequences may have lifelong sequelae. This possibility has extraordinary scope given that the prevalence of depression more than doubles during the transition from childhood (2–4%) to adolescence (4% to 14%)^2,14,15^, and at least half of the individuals who develop depression in their lifetimes experience their first depressive episode by age 14, and three-fourths by age 24^16^.

Early adolescence may be a more stressful time for females than males due to the cooccurrence of multiple stressors. Early maturing girls are especially vulnerable because of shifts in body image and the increased likelihood of being sexualized or harassed^17,18^. Symptom presentation differs between adolescent females and males, with females being more likely to display internalizing problems (e.g., self-blame, social withdrawal) while males typically show more externalizing behaviors (e.g., aggression, substance abuse)^19,20^. Females are more likely to attempt suicide, while males are more likely to complete suicide due to the lethality of the methods used^21^. These differences in symptom presentation may lead to differential sequelae in adulthood.

For effective policy and intervention, there remains much to know about the adulthood functioning of individuals who experienced depression during adolescence. One recent seminal study followed a sample of 1420 youth from age 9 to age 30 and assessed for psychiatric disorders up to 8 times between the ages of 9 and 16 years^22^. They found that depression during childhood or adolescence was associated with higher risk of anxiety, illicit substance use disorders, worse health, and poorer social functioning in adulthood^22^. Further, a meta-analysis published in 2019 reviewed 31 articles of 24 different cohorts and reported consistent associations between depression during adolescence and a wide range of negative functional outcomes an average of 9 years later, notably lower academic achievement and higher rates of unemployment^23^. Taken together, there is clear evidence that experiencing depression during adolescence has broad implications for adulthood functioning, though no studies to our knowledge have examined this association beyond age 35, or addressed the potential modifiability of these long-term sequelae. Indeed, efforts to develop effective depression programs in adolescence have been promising^24–29^, though their effects have seldom been followed for as long as 1 year. Therefore, we have little evidence to inform whether effective treatment for depression during adolescence would also confer benefits for functioning in adulthood.

The present study sought to extend these gaps in our knowledge by leveraging data from the National Comorbidity Survey Replication (NCS-R), and compared adulthood functioning in nine domains between individuals with a history of depression that began during adolescence (between the ages of 10 to 20) to those whose first episode occurred in adulthood (age 21 and older). Consistent with theoretical models of adolescence as a sensitive period of development, we hypothesized that adolescent-onset participants would be less likely to reach social milestones (e.g., marriage, children), more likely to have poorer occupational outcomes (e.g., income, education), and more likely to have poorer health (e.g., obesity). We further hypothesized these patterns would differ when looking at females and males separately, and would be less robust among participants who received treatment for their depression during adolescence.

## Method

### Participants

The present study included 3360 adults (67.2% female) who participated in the National Comorbidity Survey Replication (NCS-R) and experienced a major depressive episode at some point in their life. Participants were 22.8% African American, 7.5% Asian, 47.5% Caucasian, and 19.9% Latino. The National Comorbidity Survey (NCS) was a survey of English-speaking household residents in the United States above the age of 18 designed to collect nationally representative data on the prevalence, risk factors, and consequences of psychiatric disorders within the United States^30^. The most recent replication, NCS-R (*n* = 9282), was completed between February 2001 and April 2003 and reports on the prevalence and correlates of mental disorders with a new nationally representative household survey. The NCS-R includes assessments based on the Diagnostic and Statistical Manual of Mental Disorders, Fourth Edition (DSM-IV) diagnostic criteria^30,31^. All study procedures were approved by the Human Subjects Committees of both Harvard Medical School and the University of Michigan and all methods were performed in accordance with the relevant institutional and legal guidelines and regulations.

### Procedures

Participants in the NCS-R were recruited using a multi-stage clustered area probability sample of households, returning a 70.9% response rate and a nationally representative sample of English-speaking adults (aged 18 or older) living in non-institutionalized civilian households within the contiguous United States^32^. All participants provided informed consent to participate. Respondents completed surveys using computer-assisted personal interview (CAPI) methods in their own home, administered by trained interviewers.

### Measures

#### Depression onset

Diagnostic assessments were completed in a face-to-face structured survey interview. Participants were administered the version of the World Health Organization (WHO) Composite International Diagnostic Instrument (CIDI) made for the World Mental Health (WMH) Survey Initiative, which included all the major classes of psychiatric disorders^33^. The interview was conducted in two parts. Part I was administered to all participants and focused on assessing mood disorders, anxiety disorders, behavior disorders, eating disorders, and substance use disorders. Part II was administered only to participants who (1) met lifetime criteria for any of the disorders assessed in Part I, (2) met subthreshold lifetime criteria for any disorder and received treatment for one of them, or (3) ever made a plan to commit suicide or attempted suicide^32^. CIDI diagnoses and diagnoses based on the Structured Clinical Interview for DSM-IV (SCID) showed good levels of consistency in a probability subsample for NCS-R participants for mood disorders^34^. For the present study, it was important to examine the age at which participants experienced their first episode of MDD. Participants were categorized as having “adolescent-onset” depression if they experienced their first episode of MDD between 10 and 20 years of age. The NCS-R study asked participants to indicate how old they were when they experienced their first episode of MDD. Participants reported depression onset between the ages of 4 and 89 years old. Individuals (*n* = 170) whose first depressive episode occurred before the age of 10 were excluded from analyses.

#### Adult functional outcomes

The following adult functional outcomes were assessed via self-report on the face-to-face interview: marital status, employment history, number of biological children, household income, years of education, receipt of welfare assistance, arrests, and current obesity. Marital status was measured categorically with three options for participants to choose from: (1) “married/cohabiting”, (2) “divorced/separated/widowed”, and (3) “never married”. Their responses were then recoded as a dichotomous outcome; 1 “never married” vs 0 for all other responses. Participants who had been married also provided the “# [of] marriages ended in divorce/annulment”, which was recoded into 1 “ever divorced” or 0 for “never divorced”.

Employment was measured categorically, with three options for participants to indicate their “work status”: (1) “employed”, (2) “unemployed”, and (3) “not in labor force” which was recoded for analyses into 1 “unemployed” vs 0 all other responses. Participants’ self-report of the number of children was recoded dichotomously for the analysis: 1 “no children” vs 0 for all other responses. Participants reported their years of education in the following categories: (0) “0–11 years”, (1) “12 years”, (2) “13–15 years”, and (3) “greater than or equal to 16 years”. Annual household income was measured continuously from $0 to $200,000 or greater, which was then recoded into quartiles for analysis: (0) lowest–17,499, (1) $18,000–$37,999, (2) $38,000–$69,999, (3) 70,000 or higher. Receipt of welfare was measured dichotomously based on participants 1 “yes” or 0 “no” responses to the question, “Have you ever received public assistance or welfare since turning age 18—such as Aid to Families with Dependent Children, General Assistance, or Temporary Assistance for Needy Families?” Participants also responded with 0 “true” or 1 “false” to whether they had “never been arrested.” Obesity was measured categorically, with individuals with a BMI > 29.9 categorized as 1 “obese” vs 0 all other BMI categories.

#### Treatment

Participants were asked to indicate their answer (in years) to, “How old were you the first time you got helpful/effective treatment for your sadness/discouragement/lack of interest?”. Responses ranged from 0 to 79 years (mean = 32.45 ± 14.35). Responses were recoded into a dichotomous variable such that values between 9 and 21 were coded as 1 ‘received helpful treatment during adolescence’ or 0 ‘did not receive helpful treatment during adolescence’.

#### Covariates

The present analyses controlled for age, gender, ethnic minority status, current MDD at the time of assessment, and the total number of years participants experienced at least one depressive episode. Current MDD was indexed by the occurrence of a major depressive episode (MDE) in the past 12 months. To obtain the total number of depressed years, the NCS-R survey asked participants, “how many different years in your life did you have at least one episode?”.

### Data analysis

The datasets analyzed for the current study are publicly available in the Inter-university Consortium for Political and Social Research (ICPSR) repository at the Institute for Social Research at the University of Michigan, https://www.icpsr.umich.edu/web/ICPSR/studies/20240. Logistic (for binary outcomes such as marital status) and ordinal (for non-dichotomous categorical variables such as household income) regression models were used to look at differences in the odds of each adult outcome for participants with adolescent-onset relative to adulthood-onset depression status. All analyses were conducted in SPSS Version 28 using ordinal regression within the PLUM command and employed a logit link function. Positive coefficient estimates indicated higher log odds probabilities of the higher values for each outcome. Models for each outcome were conducted unadjusted, then adjusted for key covariates, then stratified by gender and self-reported treatment during adolescence. Model fit was determined by comparing −2 log likelihood of the adjusted models to the intercept only models using *Χ*^2^, and further evaluated using Pearson *Χ*^2^ tests of goodness-of-fit to the data. For all outcomes, adolescent-onset depression improved model fit relative to the intercept only models, all *p* < 0.001, and the predictors were a good fit to the data, all *p*s > 0.19. Some participants had missing data for number of children and employment status, therefore multiple imputation was used to maximize available data and the results from the pooled, imputed data across 5 imputations are reported. Post-hoc moderation analyses were conducted using the PROCESS Macro for SPSS^35^ where coefficient estimates for dichotomous outcomes were expressed in log-odds but are otherwise unstandardized coefficient estimates. Results should be considered statistically significant when *p* < 0.05, and a *p* < 0.006 corrects for multiple comparisons across the nine outcomes.

## Results

Adolescent-onset depression occurred among approximately one third (34.9%) of NCS-R participants with any history of MDD. Table 1 summarizes participant characteristics across all study variables.

**Table 1:** Characteristics of NCS participants with a history of depression (*n* = 3360).

	*M *(*SD*)	*% *(*n*)
**Age**	41.75 (14.66)	
**Female**		67.2 (2258)
**Race/ethnicity**		
Asian		7.5 (252)
Hispanic/Latin		19.9 (667)
Non-Hispanic white		47.5 (1596)
Black		22.8 (767)
**Current MDD (past 12 months)**		31.1 (1046)
**Age of first MDD onset**	28.50 (13.24)	
**Adolescent-onset MDD**		34.9 (1173)
**Helpful treatment during adolescence**		1.4 (48)
**Years of education**		
0–11 years		17.8 (599)
12 years		28.2 (947)
13–15 years		28.8 (967)
16+ years		25.2 (847)
**Marital status**		
Married/cohabitating		48.1 (1,615)
Divorced/widowed		27.2 (915)
Never married		24.7 (830)
**Number of living children**	1.82 (1.53)	
**No children**		7.0 (898)
**Currently unemployed**		63.8 (234)
**Household income**	$52,425.16 ($47,073.25)	

**Figure 1:**
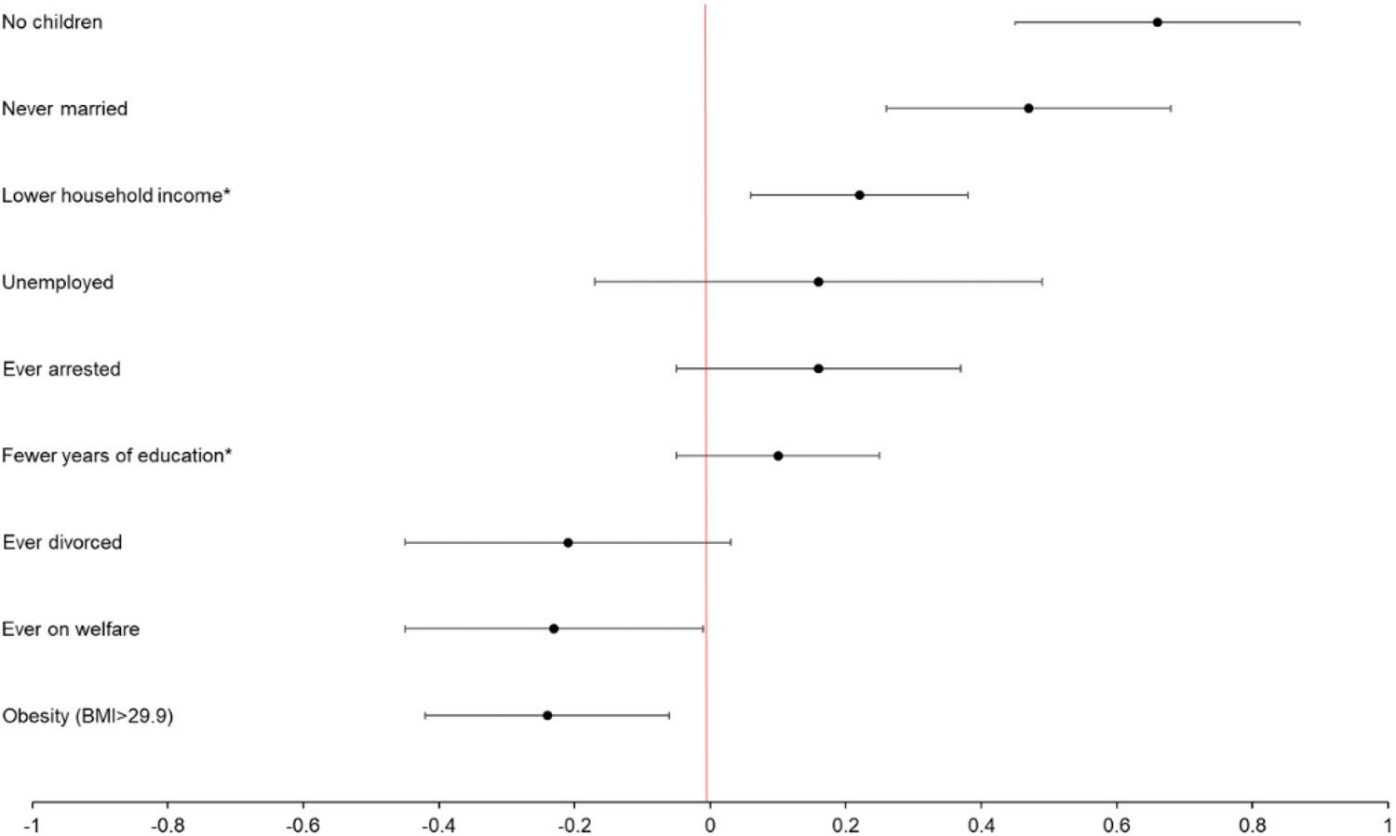
Estimated odds of functional outcomes by adolescent relative to adulthood-onset MDD.

Rows 1 and 2 of Table 2 provide the unadjusted and adjusted estimates for the association between adolescent-onset MDD and each outcome relative to adulthood-onset MDD. Individuals who experienced their first depressive episode during adolescence were less likely than those whose first episode occurred in adulthood to get married, have children, had lower household incomes, but were also less likely to ever be on welfare and less likely to be obese. These associations were robust after controlling for age, gender, race, ethnicity, whether they were currently depressed at the time of the study, and the number of years they had been depressed in their lifetime. Figure 1 displays the estimated effect of adolescent-onset, relative to adulthood-onset, depression for each functional outcome in adulthood.

**Table 2:** Estimated associations between depression during adolescence and social outcomes in adulthood.

	Dependent variable																	
	Never married		Ever divorced		Unemployed		No children		Household income (quartile)		Years of education		Ever on welfare as adult		Ever arrested		Obesity (BMI ≥ 30)	
	Est. (SE)	95% CI	Est. (SE)	95% CI	Est. (SE)	95% CI	Est. (SE)	95% CI	Est. (SE)	95% CI	Est. (SE)	95% CI	Est. (SE)	95% CI	Est. (SE)	95% CI	Est. (SE)	95% CI
1. Unadjusted	1.28 (0.083)***	1.11–1.44	−0.62 (0.11)***	−0.83 to 0.41	0.21 (0.14)	−0.06 to 0.49	1.15 (0.10)***	**0.95–1.35**	−0.14 (0.07)*	−0.28 to 0.01	0.01 (0.07)	−0.12 to 0.14	−0.05 (0.09)	−0.23 to 0.13	0.30 (0.09)***	**0.13–0.48**	−0.22 (0.08)**	**−0.39 to 0.06**
2. Adjusted	0.47 (0.10)***	**0.27–0.68**	−0.21 (0.12)^+^	−0.45 to 0.03	0.16 (0.17)	−.016 to 0.49	0.66 (0.10)***	**0.46–0.87**	−0.22 (0.08)**	**−0.38 to 0.06**	−0.10 (0.08)	−0.25 to 0.05	**−0.23 (0.11)**	−0.45 to 0.01*	0.16 (0.11)	−0.06 to 0.37	−0.24 (0.09)**	**−0.43 to 0.06**
3. Females	0.44 (0.13)***	**0.20–0.69**	−0.08 (0.15)	−0.37 to 0.22	0.24 (0.19)	−0.14 to 0.61	0.73 (0.13)***	**0.47–0.98**	−0.29 (0.10)**	**−0.47 to 0.10**	−0.001 (0.09)	−0.19 to 0.18	−0.21 (0.12)	−0.45 to 0.04^+^	0.22 (0.14)	−0.06 to 0.49	−0.20 (0.12)^+^	−0.42 to 0.03
4. Males	0.54 (0.18)**	**0.19–0.89**	−0.48 (0.22)*	**−0.91 to 0.06**	−0.06 (0.32)	−0.70 to 0.58	0.58 (0.16)***	**0.26–0.90**	−0.13 (0.14)	−0.40 to 0.14	−0.29 (0.13)*	**−0.53 to 0.04**	−0.29 (0.25)	−0.77 to 0.19	0.09 (0.17)	−0.25 to 0.42	−0.39 (0.17)*	**−0.72 to 0.05**
5. No treatment	0.50 (0.11)***	**0.30–0.71**	−0.20 (0.12)	−0.44 to 0.05	0.20 (0.17)	−0.13 to 0.52	0.66 (0.11)***	**0.45–0.87**	−0.24 (0.08)**	**−0.40 to 0.08**	−0.11 (0.08)	−0.26 to 0.05	−0.21 (0.11)	−0.43 to 0.01^+^	0.16 (0.11)	−0.06 to 0.37	−0.23 (0.09)*	**−0.42 to 0.049**
6. Treatment	−1.02 (1.10)	−3.18 to 1.14	0.41 (1.93)	−3.37 to 4.19	−2.84 (1.73)	−6.24 to 0.56	0.08 (0.90)	−1.70 to 1.85	0.25 (0.73)	−1.18 to 1.68	0.98 (0.75)	−0.50 to 2.46	−1.66 (1.36)	−4.32 to 1.0	–	–	−0.28 (0.90)	−2.04 to 1.48
7. Early adolescence (10–13 yrs)	0.52 (0.16)***	**0.20–0.84**	−0.34 (0.20) +	−0.73 to 0.06	−0.07 (0.29)	−0.64 to 0.50	0.77 (0.16)***	**0.46–1.09**	−0.32 (0.13)*	**−0.56 to 0.07**	0.04 (0.12)	−0.21 to 0.28	−0.07 (0.18)	−0.42 to 0.27	0.22 (0.17)	−0.12 to 0.55	−0.17 (0.15)	−0.47 to 0.13
8. Middle adolescence (14–17)	0.55 (0.14)***	**0.27–0.83**	−0.57 (0.18)**	**−0.93 to 0.22**	0.23 (0.22)	−0.20 to 0.67	0.73 (0.15)***	**0.42–1.03**	−0.24 (0.11)	−0.45 to 0.03	−0.17 (0.10) +	−0.38 to 0.03	−0.20 (0.16)	−0.51 to 0.11	0.07 (0.15)	−0.28 to 0.36	−0.30 (0.13)*	**−0.56 to 0.05**
9. Late adolescence (18–20)	0.50 (0.15)***	**0.21–0.78**	−0.22 (0.17)	−0.57 to 0.12	0.18 (0.23)	−0.27 to 0.62	0.62 (0.14)***	**0.35–0.89**	−0.34 (0.11)**	**−0.56 to 0.11**	−0.09 (0.11)	−0.30 to 0.12	−0.14 (0.16)	−0.45 to 0.18	0.35 (0.16)*	**0.04–0.65**	−0.28 (0.14)*	**−0.55 to 0.02**
10. Emerging adulthood (21–24)	0.16 (0.15)	−0.14 to 0.45	0.03 (0.17)	−0.30 to 0.36	−0.06 (0.25)	−0.55 to 0.43	0.13 (0.16)	−0.018 to 0.44	−0.26 (0.11)*	**−0.47 to 0.04**	0.04 (0.11)	−0.18 to 0.25	0.31 (0.15)*	**0.02–0.61**	0.18 (0.16)	−0.13 to 0.49	−0.07 (0.13)	−0.33 to 0.20

***p < .001, **p < .01, *p < .05, ^+^*p* < .10; bold typeface indicates statistical significance.

### Adolescent−onset MDD and adult functional outcomes by gender

**Figure 2:**
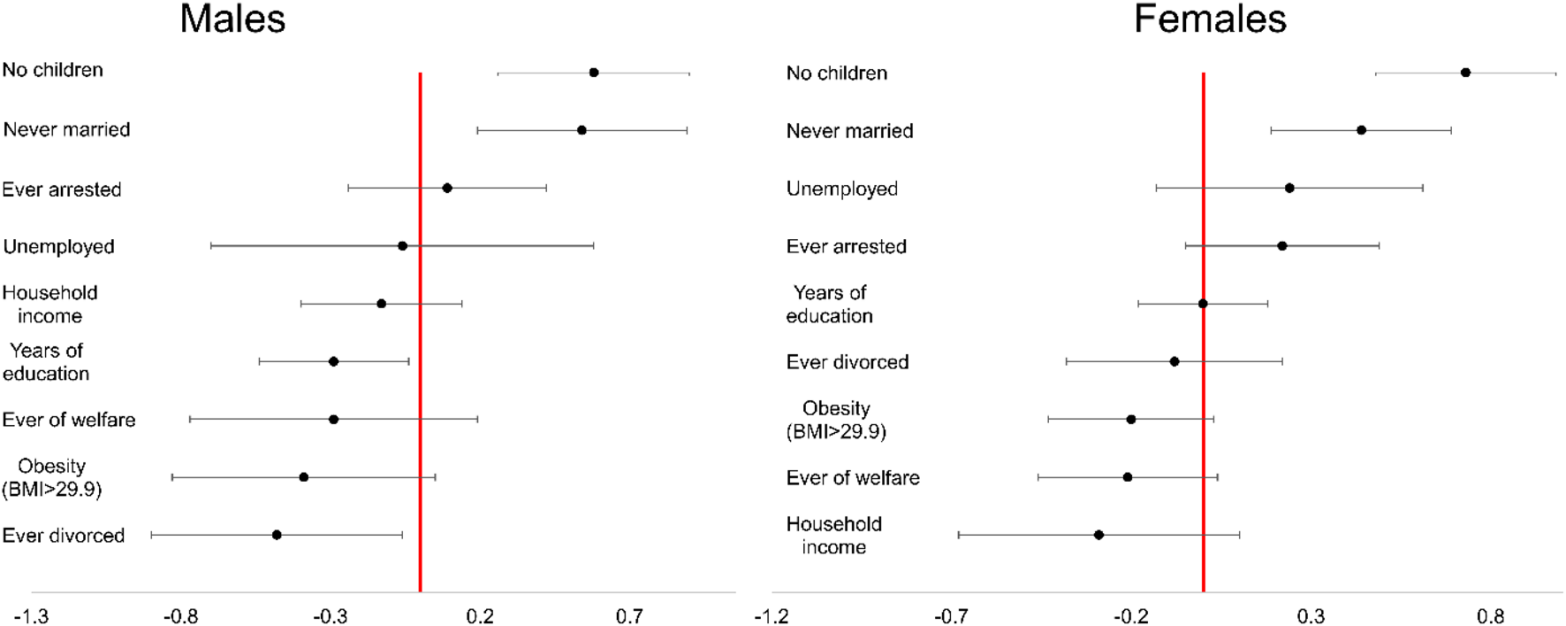
Estimated effect of adolescent-onset depression within males (left) and females (right).

Rows 3 and 4 of Table 2 provide the adjusted estimates for the association between adolescent-onset MDD and each of our outcomes stratified by gender. Figure 2 displays the estimated effect of adolescent-onset, relative to adulthood-onset, depression for each adulthood functional outcome separately within males and females. Among female participants, adolescent-onset depression was associated with not getting married, not having children, and having a lower household income. Among male participants, adolescent-onset depression was associated with not getting married, being less likely to get divorced once married, not having children, lower educational achievement, and lower likelihood of obesity.

### Treatment for adolescent-onset MDD

Only a very small proportion of the sample (1.4%) reported they received “helpful” treatment for their distress during adolescence. The association between adolescent-onset depression relative to adulthood-onset was not associated with any functional outcomes among participants who reported receiving treatment. However, among participants who did not report having helpful treatment during adolescence, those with adolescent-onset depression were less likely to be married, have children, had lower household incomes, but were less likely to be obese. Rows 5 and 6 of Table 2 provide the adjusted estimates for participants who did and did not receive helpful treatment during adolescence separately.

### Timing of adolescent-onset MDD

Among participants who experienced MDD during adolescence, age of onset was further categorized into early (10–13), middle (14–17), and late adolescence (18–20). The prevalence of depression onset across these groups was 9% early (*n* = 307), 13.8% middle (*n* = 465), 11.9% late adolescence (*n* = 401), 10.7% emerging adulthood (*n* = 359), and 54.5% adulthood (*n* = 1828). Rows 7–10 of Table 2 include the adjusted odds of each outcome by early, middle, late, and emerging adulthood MDD onset compared with adult onset. Relative to individuals with adult-onset MDD, individuals with onset during early adolescence were less likely to have married, have children, and had lower household incomes. Relative to individuals with adult-onset MDD, individuals with onset during middle adolescence were less likely to marry, have children, divorce, and be obese. Relative to individuals with adult-onset MDD, individuals with onset during late adolescence were less likely to marry, have children, and be obese, but more likely to have lower household incomes and have been arrested.

### Post-hoc analyses

Given the pattern of outcomes observed for males and females separately, and for adolescents whose depression onset at different ages, we conducted a series of post-hoc analyses testing whether gender or age of depression onset within adolescence moderated the association between adolescent-onset depression and each outcome.

Gender was a non-significant moderator when predicting never having been married, β = 0.47 (*SE* = 0.25), *p* = 0.06, divorce, β = −0.53 (*SE* = 0.29), *p* = 0.07, educational attainment, b = −0.19 (*SE* = 0.10), *p* = 0.06, and welfare, β = −0.53 (*SE* = 0.29), *p* = 0.07. Gender did not moderate the effect of adolescent-onset depression on unemployment, β = −0.21 (*SE* = 0.42), *p* = 0.61, not having children, β = 0.07 (*SE* = 0.24), *p* = 0.77, household income, *b* = 0.02 (*SE* = 0.11), *p* = 0.88, being arrested, β = −0.34 (*SE* = 0.24), *p* = 0.17, or obesity, β = −0.13 (*SE* = 0.23), *p* = 0.57.

Age of depression onset within adolescence was a non-significant moderator of the effect of depression on welfare use, β = −0.41 (*SE* = 0.22), *p* = 0.06. Age of depression onset within adolescence did not moderate the effect of depression on never having been married, β = 0.22 (*SE* = 0.23), *p* = 0.33, divorce, β = −0.13 (*SE* = 0.25), *p* = 0.62, educational attainment, b = 0.02 (*SE* = 0.09), *p* = 0.80, β = −0.07 (*SE* = 0.35), *p* = 0.84, not having children, β = 0.22 (*SE* = 0.23), *p* = 0.33, household income, *b* = 0.08 (*SE* = 0.10), *p* = 0.43, being arrested, β = −0.26 (*SE* = 0.23), *p* = 0.28, or obesity, β = 0.08 (*SE* = 0.20), *p* = 0.69.

## Discussion

Adolescence is often posited as a sensitive period of social development, during which profound changes to behavior and affect such as those experienced during a depressive episode should lead to long-lasting changes to functioning. The present study provided clear and specific evidence to support this, such that participants who experienced their first depressive episode during adolescence, relative to those who experienced depression for the first time in adulthood, were more likely to be unmarried, not have children, and have lower household incomes in adulthood, even after accounting for depression recurrence, age, gender, race, and ethnicity. These patterns of association did not significantly differ by gender or age of depression onset within adolescence. However, stratified analyses by gender suggest that when comparing across outcomes, adolescent-onset depression was associated with some overlapping (i.e., less likely to marry or have children) but some diverging outcomes in adulthood (i.e. lower household income quartile for women and less educational attainment for men). Importantly, none of these associations were observed among participants who reported receiving helpful treatment during adolescence. These observations underscore the idea that adolescence is a sensitive period for social development, during which MDD may lead to cumulative long-term social impacts. Efforts to effectively treat adolescent depression may therefore have broader implications on society and social development that extend beyond mental health.

Individuals who experienced their first depressive episode during adolescence were less likely to marry, have children, and reported lower household incomes compared to individuals who experienced their first depressive episode during adulthood. The gestalt of these findings were consistent with the existing literature that adolescent-onset depression is associated with poorer adulthood functioning in social, health, and financial/educational domains^22,23^. Outcome-by-outcome comparisons across these studies are somewhat difficult to make because many past studies created composite indices for these domains, rather than looking at outcomes individually. Yet, a close look at the discrepancies in our findings highlight important ways in which the present data extend our knowledge. First, until the present study, no study has looked at associations between adolescent-onset depression and adulthood functional outcomes past the age of 35; on average adulthood outcomes were assessed at age 24^23^. Given that our sample was 42 ± 15 years, many functional outcomes take on greater meaning. Specifically, the meta-analysis of 31 studies on this topic (in which participants were an average of 24 years) observed no association between adolescent-onset depression and marital/relationship status^23^, while one study conducted in a 30-year-old sample observed a negative association between adolescent-onset depression and adulthood marital status^22^. However, it is not uncommon in industrialized nations for individuals to get married after age 30. Assessment of these social outcomes (e.g., marriage, children, divorce) in a developmental phase when the mode age for these milestones has passed offers a more interpretable test of the theory that adolescence is a sensitive period of social development that can be disrupted by MDD.

The present study also responded to an important call to action^23^, namely the need for clarity on the role of gender. Importantly, gender did not moderate the association between adolescent-onset depression and any outcome. This suggests that the potential effect of experiencing a depressive episode during adolescents on adulthood functioning a similar for males and females. Notably, however, stratified analyses indicated that some outcomes were more relevant to one gender than the other. Within females, adolescent-onset depression was related to lower household income, whereas within males adolescent-onset depression was related to lower educational attainment. Previous studies have reported that adolescent-onset depression was associated with lower past-year income^23^ and worse financial outcomes overall^22^. Our observations extend these findings by suggesting that the pathway to worse financial outcomes may differ for adolescent males and females, such that for females income may be more relevant than educational attainment while the inverse may be more relevant for males. This may justify the addition of gender-specific modules in adolescent treatment programs that focus on applying skills learned in the program to social and occupational goals.

We also examined the question of developmental sensitivity within adolescence as well. We examined the association between adolescent-onset depression and risk for each adulthood outcome between early, middle, and late adolescence as well as within each of these age groups separately. Given the recurrent nature of depression as well as the multifactorial human development that occurs in adolescence, we expected individuals with onset reported in early adolescence might be the most vulnerable to the potential long-term consequences of the illness. Yet, this hypothesis was not confirmed. Importantly, the age of depression onset during adolescence did not moderate the association between experiencing depression during adolescence overall and any adulthood outcomes. However, the outcomes associated with adolescent- relative to adulthood-onset depression varied within these onset groupings. Depression onset during late adolescence was associated with the largest number of, and perhaps the most consequential (e.g., arrests), outcomes. This observation may inspire optimism that effective treatment during emerging adulthood or prevention during high school may be ideal contexts in which to mitigate the broader sequelae of adolescent-onset depression. Further, poorer adulthood functioning among participants with depression onset between ages 18–20 may reflect the protective nature of schools, families, and communities of origin during earlier stages of adolescence in mitigating the broader impacts of adolescent-onset depression which could be leveraged for treatment.

The conclusions of this study should be considered in the context of its strengths and limitations. First, this data is cross-sectional and participants were asked to retrospectively recall disorders and personal life events. We cannot infer whether depression leads to these outcomes in adulthood or which third variables are at play, though the similar pattern of results with prospective, longitudinal observations provide compelling convergent validity^22^. Second, participants in this sample could have been adolescents between 1921 and 1996, but most were adolescents in the 1960s and 1970s. Since then, both recognition and prevalence of depression among adolescents has increased^36,37^, and social contexts (e.g., digital and social media, academic expectations, among others) during adolescence have changed. Attention to whether generational differences in social contexts around adolescence mitigate or exacerbate the long-term sequelae of depression across the lifespan are needed, particularly to implement effective policy. Third, individuals who had childhood-onset depression were excluded from our analyses (*n* = 170). The rationale was that only data on age of onset for a first episode was available, not each subsequent episode; it is unknown which individuals with childhood-onset depression also had depression during adolescence. However, given the known chronic and recurrent course of depression^38,39^, it’s likely that many of these individuals also experienced depressive episodes during adolescence, and may therefore be susceptible to the developmental sensitivity being tested here. Third, there are many approaches to defining adolescence; 10–24 has been described as the “adolescence window” due to the increased importance of social development^13^ whereas other studies limit the range to 13 to 17.9 years^40^. Our age range (10–20 years) was inclusive of puberty while also adhering to policy that regards age 21 as a meaningful social and legal transition to adulthood in many respects. Furthermore, socioeconomic status (SES) in childhood has been linked to risk for psychopathology in childhood and adolescence^41^. The NCS-R assessed childhood SES with one item that asked about receipt of public assistance while growing up. While our results remain unchanged when accounting for this variable, only 5.3% of the sample endorsed the item and the low frequency, categorical nature of the variable was unlikely to reflect all of the aspects of childhood SES recognized as important in developmental psychopathology.

Despite these limitations, the present study contributes to a growing literature documenting the breadth of sequelae that follow depressive episodes during adolescence and the potential benefit of directing more resources to their treatment and even prevention. Indeed, while this study focused on categorically comparing individuals with and without adolescent-onset depression, even subthreshold depressive symptoms have implications for functioning^42^. Cognitive-behavioral programs are effective at reducing adolescents’ depressive symptoms in the short term^24,26,27,29,43,44^, and can be delivered in school-based settings^25,45^. Further, interventions targeting parenting behaviors are associated with improved outcomes for youth generally^46^, as well as depressive and internalizing symptoms specifically^47^. This at least means that many interventions show promise in mitigating or even delaying the onset of depression. However, given that late-adolescence was associated with the largest number of adulthood outcomes, attention to delaying depression past this period (ages 18–20) must be given. Further, the programs thus far have focused on measuring symptom severity post-intervention rather than broader social outcomes whose measurement may illuminate causal trajectories between adolescent-onset depression and functional outcomes in adulthood.

## Data Availability

The datasets analyzed for the current study are publicly available in the Inter-university Consortium for Political and Social Research (ICPSR) repository at the Institute for Social Research at the University of Michigan, https://www.icpsr.umich.edu/web/ICPSR/studies/20240.
